# Diversity Among Academic Orthopaedic Surgery Sports Medicine Faculty Across US Residency Programs

**DOI:** 10.5435/JAAOSGlobal-D-26-00085

**Published:** 2026-09-08

**Authors:** Terence L. Thomas, Alexander P. Zavitsanos, Claude J. Regis, John Czarnicki, Harrison S. Fellheimer, Gabriel Onor, William L. Justice, Jalen Broome, Damon V. Briggs, Edward S. Chang, Meghan Bishop, Christopher C. Dodson

**Affiliations:** From the Rothman Orthopaedic Institute at Thomas Jefferson University (Dr. Thomas, Dr. Onor, Dr. Justice, Dr. Broome, Dr. Briggs, Dr. Chang, Dr. Bishop, Dr. Dodson), Philadelphia, PA; the Sidney Kimmel Medical College (Zavitsanos, Regis, Czarnicki, Fellheimer), Thomas Jefferson University, Philadelphia, PA, and the Inova Sports Medicine (Dr. Chang), Fairfax, VA.

## Abstract

**Background:**

Despite growing attention to workforce diversity in orthopaedic surgery, limited data exist describing the demographic composition of academic sports medicine faculty. This study examines the diversity of sports medicine fellowship-trained orthopaedic surgery faculty across US orthopaedic surgery residency training programs.

**Methods:**

A cross-sectional analysis of all ACGME-accredited orthopaedic surgery sports medicine faculty was conducted. Faculty data included sex, race, level of professorship, residency attended, year of residency graduation, and h-index. Underrepresented in Orthopaedics (URiO) was defined as non-White and non-Asian by race and all women. Descriptive statistics were performed.

**Results:**

A total of 812 sports medicine-fellowship trained faculty were included in the analysis: 91% men and 9% women. Racial distribution of faculty was 83% White, 11% Asian, 4% African American, and 1% Hispanic. The region of the United States with the greatest percentage of URiO faculty was the South Atlantic (26%). Most URiO faculty graduated from residency in the last quarter century: 42% from 2012 to 2025 and 41% from 2001 to 2011. No differences were observed between URiO and non-URiO with regard to academic rank or H-Index.

**Conclusions:**

The present findings highlight persistent disparities in diversity among orthopaedic sports medicine faculty. Intentional efforts remain warranted to mitigate structural barriers in URiO recruitment to the sports medicine subspecialty and orthopaedic surgery as a whole.

Diversity within healthcare teams is widely recognized as essential to improving outcomes, strengthening communication, and advancing equity in patient care.^1^ A more representative workforce has been linked to greater patient trust and satisfaction, enhanced therapeutic alliances, and better adherence to recommended treatment, particularly among populations that have historically experienced disparities in access and outcomes.^1-4^ Beyond the clinical encounter, diverse academic environments can expand the range of perspectives that inform research questions, educational priorities, and health system policies, thereby shaping innovation and care delivery in ways that better meet the needs of heterogeneous patient populations.^1-4^

Concordance between patients and physicians—whether by race/ethnicity or sex—has also been associated with improved experience and measurable differences in process and outcome metrics. Studies across multiple settings describe higher satisfaction, greater participation in shared decision making, and improved communication when patients receive care from clinicians who share salient identity characteristics, with some analyses also noting lower utilization barriers and better follow-up among minoritized groups in concordant dyads.^5-11^ Although concordance is not a panacea for structural inequities, these observations underscore how representation within the physician workforce can influence care at the point of service while contributing to longer-term trust in medical institutions.

Orthopaedic surgery continues to lag behind many other specialties in the representation of women and racially/ethnically underrepresented groups across faculty, leadership, and the training continuum. Despite incremental gains in certain areas, persistent gaps remain in academic ranks and program leadership, which has implications for mentorship, sponsorship, and visibility of role models for trainees considering subspecialty careers.^12,13^ These disparities occur in parallel with the growing need for musculoskeletal care among an expanding population and the broad demographic diversity of patients receiving orthopaedic services.^12,13^

Sports medicine, as a high-visibility orthopaedic subspecialty with substantial overlap across age groups, activity levels, and athletic identities, provides a salient context in which to examine how workforce diversity relates to training pipelines. Prior subspecialty reports in orthopaedics have described representation patterns among academic faculty,^14-16^ yet there are limited data specifically exploring the composition of sports medicine faculty. Addressing this gap, this study aims to assess the diversity of sports medicine faculty at ACGME-accredited orthopaedic programs.

## Methods

A cross-sectional analysis of US orthopaedic residency programs accredited by the Accreditation Council for Graduate Medical Education (ACGME) was conducted. Using publicly available data from the Doximity database, program websites, and professional profiles, active program faculty with fellowship training in surgical sports medicine were identified. Faculty sex, race and ethnicity, academic rank, residency program and graduation year, fellowship program and graduation year, and H-index were extracted. Methodology for faculty and resident alumni identification and evaluation followed approach used in a prior subspecialty diversity study within orthopaedic spine surgery.^16^ As such, race and ethnicity were collected by three independent reviewers using visual and in-text information from hospital websites and Doximity profiles. A fourth reviewer was used to resolve any conflicts or uncertainties regarding race/ethnicity and sex. The present study group comprised a diverse group of reviewers to reduce bias on the bases of race and ethnicity categories.

Data summarized for race and ethnicity were categorized based on the Association of American Medical Colleges (AAMC) guidelines.^17^ Based on the AAMC definition, racially underrepresented in medicine includes all non-White and non-Asian individuals. As women make up less than 10% of all orthopaedic attendings and only 20% of all orthopaedic residents,^18^ all women from our cohort were also considered underrepresented. Therefore, faculty were determined to be underrepresented in orthopaedics (URiO) based on the following criteria: racial and ethnic groups underrepresented in medicine by AAMC criteria (non-White and non-Asian) and all women regardless of race or ethnicity. Moreover, we also subcategorized URiO by race only which included individuals from AAMC underrepresented racial and ethnic groups, regardless of sex.

Descriptive statistics were performed to evaluate overall faculty demographics and within strata of region, academic rank, residency graduation era, and H-index. All continuous variables were compared using independent sample t-tests or Mann-Whitney U-tests. Categorical variables were analyzed with Pearson chi-square or Fisher exact tests. Statistical analyses were performed using IBM SPSS (version 29.0.0.0 (241)). Statistical significance was set at *P* < 0.05.

## Results

Table 1 details the demographics distribution of orthopaedic surgery sports medicine faculty. Across the 807 sports medicine–trained residency faculty, men comprised 732 of 807 (91%), and women comprised 75 of 807 (9%). With respect to race and ethnicity, 670 of 807 (83%) were White, 91 of 807 (11%) were Asian, 35 of 807 (4%) were African American, and 11 of 807 (1%) were Hispanic. Using the composite designations, URiO (race + women) represented 121 of 807 (15%), and URiO (Race Only) represented 45 of 807 (6%). Taken together, these findings indicate that sports faculty within residency programs remain predominantly male and White, with comparatively limited representation of women and racial and ethnic URiO groups.

**Table 1:** Demographic Distribution of Orthopaedic Surgery Sports Medicine Faculty

Variable	N (%), N = 807
Sex	
Men	732 (91)
Women	75 (9)
Race	
White	670 (83)
Asian	91 (11)
African American	35 (4)
Hispanic	11 (1)
URiO (race + women)^a^	121 (15)
URiO (race only)^b^	45 (6)

AAMC = Association of American Medical Colleges; URiO = underrepresented in orthopaedics

a URiO (race and women) includes faculty within racial groups underrepresented in medicine (non-Asian, non-White by AAMC definition) plus all women faculty, regardless of race

b URiO (race only) includes all faculty within racial groups underrepresented in medicine (non-Asian, non-White by AAMC definition)

Table 2 compares faculty by region, level of professorship, year of residency graduation, and H-index based on URiO status. Overall, US geographic regions with the highest prevalence of URiO faculty include South Atlantic (26%), followed by Mid-Atlantic (18%) and East North Central (15%). Regional distributions of faculty did not differ significantly by URiO status (*P* > 0.05). Academic rank and H-index were similar between URiO and non-URiO faculty (*P* > 0.05). There was a noticeable increase in URiO faculty over time as most URiO faculty graduated from residency in the latest 2 decades (2012 to 2025) (42%) and 2001 to 2011 (41%), respectively (*P* < 0.05) (Table 2).

**Table 2:** Comparison of Faculty Program Region, Level of Professorship, Year of Residency Graduation, and H-Index Based on URiO Status

Variable	All (N [%]), N = 807	URiO (N [%]), N = 121	Non-URiO (N [%]), N = 686	*P*
Program Region				0.172
Pacific	86 (11)	17 (14)	69 (10)	
Mountain	38 (5)	6 (5)	32 (5)	
West North Central	47 (6)	7 (6)	40 (6)	
West South Central	59 (7)	8 (7)	51 (7)	
East North Central	163 (20)	18 (15)	145 (21)	
East South Central	45 (6)	4 (3)	41 (6)	
New England	48 (6)	8 (7)	40 (6)	
Mid Atlantic	180 (22)	22 (18)	158 (23)	
South Atlantic	141 (17)	31 (26)	110 (16)	
Level of professorship				0.348
Professor	188 (23)	24 (23)	164 (30)	
Associate Professor	159 (20)	27 (26)	132 (24)	
Assistant Professor	308 (38)	54 (51)	254 (46)	
Year of residency graduation				**0.034**
1974-1989	64 (8)	5 (4)	59 (9)	
1990-2000	146 (18)	16 (13)	130 (19)	
2001-2011	331 (41)	49 (41)	282 (41)	
2012-2025	261 (32)	51 (42)	210 (31)	
Attending H-Index^a^	14.2 (15.2)	12.9 (14.4)	14.4 (15.3)	0.301

Bold values represent statistically significance, defined as *P* value < 0.05.

URiO = underrepresented in orthopaedics.

a Mean (standard deviation).

## Discussion

Orthopaedic surgery has been identified as the least diverse medical field overall and as the only surgical specialty with a significant deficit in minority department chairs compared with other surgical and nonsurgical areas.^12,16^ This study provides the first national description of the demographic composition of sports medicine trained orthopaedic surgery faculty. Two main findings emerged from this study. First, sports medicine faculty remain prominently White and male with women comprising of 9% and underrepresented groups comprising of 6% of faculty. Second, compared with previous reports in other subspecialties such as spine and shoulder and elbow surgery, sports medicine has a modestly higher representation of women. These findings are consistent with prior analyses in orthopaedic subspecialties, which have documented persistent underrepresentation among academic faculties and only incremental change over time.^14,15^ Although sports medicine appears somewhat more diverse than spine or shoulder and elbow surgery, absolute representation remains low relative to both the US workforce and the US population. Consistent with previous literature our findings suggest that gains in faculty representation have been incremental, with higher proportions of underrepresented faculty among more recent residency cohorts.^14,15^

Several implications for residency programs may be extracted from the present findings. Strategies that increase faculty diversity should be paired with structured mentorship and sponsorship designed to connect trainees from underrepresented groups to visible role models in sports medicine. Early and repeated exposure to sports medicine rotations, research, and scholarly venues may foster belonging and make the subspecialty choice feel more attainable for URiO cohorts. Program culture and communication practices that support patient centered care and inclusive learning environments have long been associated with better experiences for diverse patient populations and may also influence trainee engagement.^1^ In parallel, strengthening representation in leadership roles—such as program directors, fellowship directors, and department chiefs—may amplify mentorship capacity and shape institutional norms in ways that support diverse trainees.^12^

Although this analysis focuses on ACGME US program-level representation of sports medicine faculty, future investigations would benefit from incorporating more granular indicators of faculty engagement and influence on URiO resident decisions to pursue careers in the sports medicine subspecialty. Variables such as leadership roles in residency and fellowship education, including program director or associate program director positions, patterns of scholarly mentorship as reflected by underrepresented trainee authorship, and measures of academic productivity may offer deeper insight into how underrepresented faculty shape trainee exposure and career decision-making. Such factors may better capture meaningful mentorship, sponsorship, and advocacy than faculty presence alone. Accordingly, this study should be viewed as a foundational, descriptive assessment that highlights the need for more detailed, individual-level analyses to clarify the mechanisms through which faculty diversity may influence subspecialty pipeline outcomes.

This study has several limitations. This is a cross-sectional, program-level investigation which has inherent shortcomings. Demographic assignments provided in this study relied on visual inspection of publicly available information by multiple reviewers, which may be subject to misclassification bias. Strict reliance on program websites, Doximity profiles to determine race, ethnicity, and sex for all faculty and resident alumni has the potential to introduce inaccuracies, as some websites may have had missing alumni data or incomplete program websites. Nonetheless, the use of a diverse group of reviewers to provide a check and balance throughout data collection aimed to reduce bias. Moreover, the cross-sectional design of this study only represents a single snapshot in time as it pertains to faculty diversity. Although it can be assumed diversity among faculty has increased over time, this could not be determined from this study alone. Similarly, the inherent limitations in data available by Doximity and residency program websites create the potential for missing data. Only faculty listed with regard to residency education were included in this analysis, and therefore, the total number of underrepresented faculty across the country in both academic and private practice settings are unknown. Finally, this study also does not explore the influence that diverse faculty may have on a URiO resident's decision to pursue a career in sports medicine. Further studies on this topic are warranted.

## Conclusions

In US orthopaedic residency programs, sports medicine-trained orthopaedic surgery faculty remain predominantly male and White, with modestly higher female representation compared with other orthopaedic subspecialties. Intentional efforts remain warranted to mitigate structural barriers in URiO recruitment to the sports medicine subspecialty and orthopaedic surgery as a whole.
